# Feasibility and therapeutical potential of local intracerebral encapsulated cell biodelivery of BDNF to *App*^*NL−G−F*^ knock-in Alzheimer mice

**DOI:** 10.1186/s13195-023-01282-x

**Published:** 2023-08-18

**Authors:** Simone Tambaro, Sumonto Mitra, Ruchi Gera, Bengt Linderoth, Lars U. Wahlberg, Taher Darreh-Shori, Homira Behbahani, Per Nilsson, Maria Eriksdotter

**Affiliations:** 1 Department of Neurobiology, Care Sciences and Society; Division of Neurogeriatrics, Center for Alzheimer Research, Karolinska Institutet, Stockholm, Sweden; 2 Department of Neurobiology, Care Sciences and Society; Division of Clinical Geriatrics, Center for Alzheimer Research, Karolinska Institutet, Huddinge, Sweden; 3 Department of Clinical Neuroscience, Section of Neurosurgery, Karolinska Institutet, Stockholm, Sweden; 4 Gloriana Therapeutics, Inc., Warren, RI USA; 5 Sinfonia Biotherapeutics AB, Huddinge, Sweden; 6 Theme Inflammation and Aging, Karolinska University Hospital, Huddinge, Sweden

**Keywords:** Encapsulated cell biodelivery (ECB), Brain-derived neurotrophic factor (BDNF), Alzheimer’s disease (AD), *App*^*NL−G−F*^ knock-in mice, Therapy, Drug delivery

## Abstract

**Background:**

Alzheimer’s disease (AD) is an age-related disease characterized by altered cognition, neuroinflammation, and neurodegeneration against which there is presently no effective cure. Brain-derived neurotrophic factor (BDNF) is a key neurotrophin involved in the learning and memory process, with a crucial role in synaptic plasticity and neuronal survival. Several findings support that a reduced BDNF expression in the human brain is associated with AD pathogenesis. BDNF has been proposed as a potential therapy for AD, but BDNF has low brain penetration. In this study, we used an innovative encapsulated cell biodelivery (ECB) device, containing genetically modified cells capable of releasing BDNF and characterized its feasibility and therapeutic effects in the novel *App* knock-in AD mouse model (*App*^*NL−G−F*^).

**Methods:**

ECB’s containing human ARPE-19 cells genetically modified to release BDNF (ECB-BDNF devices) were stereotactically implanted bilaterally into hippocampus of 3-month-old *App*^*NL−G−F*^ mice. The stability of BDNF release and its effect on AD pathology were evaluated after 1, 2-, and 4-months post-implantation by immunohistochemical and biochemical analyses. Exploratory and memory performance using elevated plus maze (EPM) and Y-maze test were performed in the 4-months treatment group. Immunological reaction towards ECB-BDNF devices were studied under ex vivo and in vivo settings.

**Results:**

The surgery and the ECB-BDNF implants were well tolerated without any signs of unwanted side effects or weight loss. ECB-BDNF devices did not induce host-mediated immune response under ex vivo set-up but showed reduced immune cell attachment when explanted 4-months post-implantation. Elevated BDNF staining around ECB-BDNF device proximity was detected after 1, 2, and 4 months treatment, but the retrieved devices showed variable BDNF release. A reduction of amyloid-β (Aβ) plaque deposition was observed around ECB-BDNF device proximity after 2-months of BDNF delivery.

**Conclusions:**

The result of this study supports the use of ECB device as a promising drug-delivery approach to locally administer BBB-impermeable factors for treating neurodegenerative conditions like AD. Optimization of the mouse-sized devices to reduce variability of BDNF release is needed to employ the ECB platform in future pre-clinical research and therapy development studies.

**Supplementary Information:**

The online version contains supplementary material available at 10.1186/s13195-023-01282-x.

## Introduction

Efficient drug delivery to the brain tissue is one of the main challenges hindering the development of effective therapies against numerous neurodegenerative diseases. Various methodological advancements over the last few decades have enabled the development of unique drug delivery methods to brain parenchyma [1]. However, long-term delivery of regenerative substances (such as neurotrophins) in a sustained and controlled manner to targeted brain regions are still under development. Although various methods had been previously employed to deliver neurotrophins, some shortcomings including permeability through blood–brain barrier (BBB) need to be solved [2]. We have recently demonstrated the feasibility, tolerability, and clinical efficacy of a technological platform, termed encapsulated cell biodelivery (ECB), in delivering the neurotrophin—nerve growth factor (NGF) to the brain of individuals with Alzheimer’s disease (AD) [3–6]. The ECB platform is a versatile, controlled, and targeted approach to deliver any protein into a specific brain region. The ECB device is designed to harbor genetically modified cell lines, which actively releases the proteinaceous drug, within a semi-permeable biologically inert membrane to avoid physical contact with the surrounding tissue. Previous studies also demonstrated the feasibility of using the ECB platform in delivering neurotrophins in pre-clinical contexts, wherein the ECB devices were adapted according to the brain size and levels of drug delivery needed [7–11].

The two principal hallmarks observed in the brains of AD patients are the extra-cellular deposits of amyloid-β (Aβ) plaques and the intracellular neurofibrillary tangles consisting of hyperphosphorylated tau [12]. AD is associated with brain inflammation, synaptic loss, neurodegeneration, and memory impairment, which all worsen with time leading to loss of patients’ cognition, independence, and, ultimately, their lives. Only a few drugs have been approved to date for the treatment of AD, including three cholinesterase inhibitors and an N-methyl-D-aspartate (NMDA)-receptor antagonist. However, these drugs have mainly symptomatic effects [13]. In addition to these medications, the first anti-Aβ antibody aducanumab which has been shown to remove Aβ plaque from the brain was approved in the USA. However, its effect on slowing cognitive decline is small and varied in phase 3 trials [14]. Recently, another anti-Aβ antibody Lecanemab showed a small but statistically significant slowing of cognitive decline with a reduction of brain amyloid in a phase 3 clinical trial [15], leading to its approval in the USA [16]. All in all, the need for a treatment which modifies, stops, or prevents AD is crucial, considering that AD has a high incidence worldwide and is estimated to affect around 150 million people by 2050 [17].

In addition to Aβ targeting therapies, substances such as neurotrophins are of therapeutic interest for AD. Brain-derived neurotrophic factor (BDNF) is a neurotrophic factor which is produced and secreted in various brain regions and in the periphery [18]. BDNF has a crucial role in regulating axonal growth, neuronal differentiation, survival, and synaptic plasticity [19–22]. Most importantly, it has been shown that BDNF promotes neuronal networking and reorganization after injury. Furthermore, BDNF can reduce microglia activation and hence modulate neuroinflammation [23, 24]. In the brain, BDNF is mainly synthesized in cell bodies of neurons and glial cells and then transported to presynaptic terminals and postsynaptic dendrites [25]. The localization of BDNF and its receptor, tropomyosin receptor kinase B (TrkB), to glutamatergic synapses regulate neurotransmitter release, ion channel activity, axonal pathfinding, and neuronal excitability [18]. The highest level of BDNF mRNA is found in the hippocampus [26]. Hippocampal BDNF expression is primarily localized in the CA2, the medial portion of CA1, and the nuclei of granule cells in the dentate gyrus and the pyramidal cell layer [27]. In addition, BDNF is mostly produced and expressed in the entorhinal cortex, a key brain area for learning and memory [28, 29].

Reduced gene expression and protein levels of BDNF have been found in AD patients’ serum and brain tissue compared with healthy controls [30–33]. Importantly, higher expression of BDNF correlates with slower cognitive decline in AD patients, which is even more pronounced with individuals displaying severe AD pathology [29]. Accordingly, it is conceivable to increase BDNF levels in the brain by directly supplementing BDNF or indirectly stimulating BDNF expression as a potential disease-modifying approach for AD. However, using BDNF as a therapeutic molecule is challenging. Systemically administered BDNF is degraded during circulation in the blood and intact BDNF cannot cross the BBB making its delivery to the brain very difficult [34]. In this study, we used a second-generation small-sized BDNF releasing implant (ECB-BDNF) developed by Gloriana Therapeutics, Inc. for sustained local release of BDNF in the brain tissue. The ECB-BDNF devices were surgically implanted into the hippocampus of the *App knock-in* AD mouse model, *App*^*NL−G−F*^. This AD mouse model expresses endogenous levels of the amyloid precursor-protein (APP) harboring the Swedish, Arctic, and Beyreuther familial AD mutations leading to robust Aβ pathology resulting in synaptic degeneration, neuroinflammation and memory impairments [35]. We evaluated the safety and tolerability of the ECB-BDNF device implantation, followed by studying the effect of BDNF delivery on cognition as well as AD-related markers.

## Methods

### Preparation of the ECB device

#### Preparation of plasmid

Preparation of the plasmid encoding BDNF has been described in detail elsewhere [7]. The plasmid pT2.CAn.hopp.BDNF, containing the entire pre-pro-BDNF sequence, was similarly generated. The sequence was codon optimized for mammalian expression (GeneArt, Regensburg, Germany) and the neomycin gene was used to allow for G418 (Sigma-Aldrich, Germany) selection of recombinant cells. For the transient expression of the sleeping beauty transposase, the separate vector pCMV-SB-100 × was used.

#### Generation of BDNF expressing cells and subsequent cell maintenance

Human retinal pigment epithelial (RPE) cell line—ARPE-19 (CRL-2302, ATCC; Manassas, VA, USA) were cultured using DMEM/F12 media containing GlutaMAX (Invitrogen, USA) supplemented with 10% heat inactivated fetal bovine serum (FBS) (Cat no. 0010, Hyclone, USA), henceforth mentioned as complete DMEM/F12 media, under standard cell culture conditions 37 °C and 5% CO_2_, respectively. ARPE-19 cells were passaged using TrypLE (Life Technologies, Carlsbad, CA, USA) after reaching 75–80% confluency until used. ARPE-19 cells were co-transfected with pT2.CAn.hopp.BDNF and pCMV-SB-100x (expressing SB transposase without Neomycin cassettes) plasmids using FuGENE (Roche, Switzerland), according to the manufacturer’s protocol. Single cells that incorporated the BDNF-neomycin expression cassette were selected using G418 (Sigma-Aldrich, USA) and were expanded clonally and further characterized for high and stable BDNF production in vitro. One clone was selected after assessing several clones for stable and long-term BDNF production post encapsulation in both human endothelial serum-free media (HE-SFM; Gibco, USA) in vitro as well as after explantation from normal rat brains.

#### Preparation of ECB-BDNF and ECB-Control devices

Semi-permeable polysulfone hollow fiber membranes (Gloriana Therapeutics, USA; 280 kDa median molecular weight cut-off) were utilized to prepare ECB-BDNF devices 3.5 mm long and 0.4 mm in diameter. The devices were threaded with polyester terephthalate (PET) yarn matrix (Swicofil, Switzerland) to support cell adhesion and then injected with 25,000 BDNF releasing cells in a total volume of 2.5 µL HE-SFM medium using a semiautomatic custom-made cell injector system (Kineteks, Rhode Island, USA). The open end of the filled devices was then sealed using a photopolymerized acrylic adhesive (Dymax, USA) and maintained in 1 mL HE-SFM medium until used for experimentation. ECB-Control devices were prepared following the methods as described above by encapsulating ARPE-19 cells without any genetic modification. Devices without any cells served as empty device control.

### Animals

In this study, we have used female wild-type C57BL/6JRj (WT) and *App*^*NL−G−F*^ knock-in mice. *App*^*NL−G−F*^ mice contain the Swedish (KM670/671NL), the Arctic (E693G), and the Beyreuther/Iberian (I716F) mutations [36]. All animal experiments were carried out under the ethical permits ID 407 approved by the Linköping animal ethical committee and 12,570–2021 and 5406–2020 approved by the Stockholm animal ethical committee. Mice were kept on 12:12 light–dark cycle with ad libitum access to food and water and were randomly selected to receive different ECB implants for various durations (please refer " Study design for ECB implantation" Section). All animals were considered for further data analysis, unless there were technical issues with brain tissue collection or ECB collection, respectively.

### RNA isolation and sequencing

RNA isolation and sequencing were performed with hippocampal tissue obtained from a separate cohort of animals and directly frozen in RNeasy Lipid Tissue Mini Kit (74,804, Qiagen) according to the manufacturer’s protocol. RNA quality (RNA integrity number, RIN) and quantity were analyzed in a Bioanalyzer 2100 (Agilent) with the Agilent RNA 6000 Nano Kit (part number 5067–1511). NEBNext Ultra II Directional RNA Library Prep Kit for Illumina (E7760S, New England Biolabs) was used to prepare the sequencing libraries, using 200 ng of total RNA. mRNA was isolated and fragmented using the NEBNext poly(A) mRNA magnetic isolation module (E7490S, New England Biolabs) and cDNA synthesized with AmPuse XP beads (A63880, Beckman Coulter). Adaptor ligation and size selection was done according to the manufacturer’s instructions. Adaptor ligated cDNA was amplified by PCR to incorporate an Illumina compatible index sequence (NEBNext Multiplex Oligos for Illumina, Dual Index Primers Set1, E7600S, New England Biolabs). Libraries were purified with AmPure XP beads, and the size distribution of the libraries was measured by Bioanalyzer 2100 using the Agilent High Sensitivity DNA Kit (part number 5067–4626). Quantification of libraries was done with the Qubit® 2.0 Fluorometer (ThermoFisher Scientific) and Qubit™ dsDNA HS Assay Kits (Q32851, Invitrogen). All 30 libraries were pooled and diluted to 3.5 nM for sequencing on one lane of a Hiseq 3000 sequencer (Illumina), using a single read 50 bp and dual indexed sequencing strategy. Raw sequence reads in FastQ format were mapped to the mouse genome (mm10) using Tophat2 with Bowtie2 option [37, 38]. Adaptor sequences were removed using trim galore prior to mapping. BAM files containing the alignment results were sorted according to their mapped positions. Raw read counts for every gene were calculated with featureCounts from Subread package [39]. Differential gene expression analysis was performed with DEseq2, where genes with raw counts were used as input [40]. Differentially expressed genes (DEGs) were identified by adjusted *p* value for multiple testing using Benjamini–Hochberg correction with false discovery rate (FDR) values less than 0.1.

### Study design for ECB implantation

The overall study was divided into two parts. The first part addressed the tolerability and feasibility of surgery and the implanted devices, which were performed using two individual cohorts of animals—cohort 1 and cohort 2, respectively. The second part was carried out to investigate the therapeutic effect of BDNF using cohort 3. All implantation procedures of ECBs were performed at 3 months of age of the animals (wild type or *App*^*NL−G−F*^), before memory impairments start in *App*^*NL−G−F*^ mice [36]. The level of BDNF release from individual ECB’s were evaluated prior to implantation as well as post-explantation.

Cohort 1: WT (*n* = 4) and *App*^*NL−G−F*^ (*n* = 3) mice were implanted with ECB-BDNF devices and sacrificed after 1 month. Age-matched unimplanted WT (*n* = 2) and *App*^*NL−G−F*^ (*n* = 2) mice were simultaneously sacrificed to serve as controls. Post sacrifice, explanted ECBs were evaluated for attached immune cells by flow cytometry and protein expression in brain tissue sections was studied using immunohistochemistry.

Cohort 2: WT (*n* = 4) and *App*^*NL−G−F*^ (*n* = 4) mice were implanted with ECB-BDNF devices and sacrificed 2 months later together with age-matched WT (*n* = 4) and *App*^*NL−G−F*^ (*n* = 4) unimplanted (control) mice. The brains were removed and analyzed by immunohistochemistry. 

Cohort 3: One group of *App*^*NL−G−F*^ (*n* = 10) mice were implanted with ECB-BDNF devices, while another group of *App*^*NL−G−F*^ (*n* = 10) mice were implanted with ECB-Control devices, respectively. A third group of WT mice (*n* = 10) without surgery served as control group. Upon completion of 3 months treatment, these animals were evaluated for anxiety-related behavior using elevated plus maze whereas memory and learning capacity was studied using Y-maze tests. Eventually, after the completion of behavioral studies, the animals were sacrificed at the completion of 4-month post-implantation and the ECBs were retrieved from their brain. ECBs were analyzed for immune cell attachment whereas brain tissues were probed by immunohistochemistry.

### Stereotactic implantation of ECB devices

Three-month-old WT and *App*^*NL−G−F*^ mice were surgically implanted with the ECB-BDNF or ECB-Control devices, as appropriate depending on the experimental cohort. Before surgery, the mice received a preoperative pain relief injection of Rimadyl (carprofen) 5 mg/kg subcutaneously. The mice were anesthetized with isoflurane and positioned in a stereotaxic frame (Stoelting, Dublin, Ireland). A midline incision was made in the scalp and two bilateral holes were drilled through the skull. The ECB devices were bilaterally implanted in the hippocampus by an implantation cannula mounted to the stereotaxic frame. The implantation coordinates with respect to Bregma were AP: 2.9, L: ± 2.6, and DV: 4.8. After the surgery, the incision was closed with absorbable sutures. The mice were injected subcutaneously with Buprenorphine (0.05–0.1 mg/kg, daily) post-surgery for pain relief. After 1, 2, or 4 months of implantation, the mice were deeply anaesthetized and perfused with PBS. The devices were retrieved and incubated at 37 °C in HE-SFM. The brains were removed and postfixed with 4% paraformaldehyde for subsequent immunohistochemistry analysis.

### Behavioral studies

#### Elevated plus-maze (EPM)

EPM was used to investigate anxiety-related behavior. Testing was performed as previously described [41]. Shortly, EPM consists of a black plexiglas apparatus of two open (25 × 5 cm) and two closed arms (25 × 5 × 5 cm), extending from a central platform (5 × 5 cm) at 60 cm from the ground. Mice were individually placed on the central platform facing an open arm, and their behavior was recorded for 5 min. An arm entry was counted when all four paws were inside the arm. The apparatus was cleaned with 70% ethanol to remove any odor cues between each session. Behavioral measures included the number of entries and duration of time spent in each partition of the elevated plus maze.

#### Y-maze

A standard Y-maze apparatus was used to measure short-term spatial memory, made of gray plastic, and consists of three compartments (36 × 15 cm) that extend from a center platform (15 × 15 × 15 cm). Each mouse was placed in one arm facing the center of the maze and then allowed to explore freely for 5 min. The apparatus was cleaned with 70% ethanol to remove any odor cues between each session. The spontaneous behavior of the mice was manually determined by dividing the number of alternations/(total entries—1).

### Flow cytometry

The immunogenicity of the devices was evaluated in an ex vivo set up. Dissected spleen from C57BL/6 J mice was dissociated and passed through 100-micron nylon mesh in incomplete RPMI (A1049101, Invitrogen, USA) to obtain single cell suspension. Splenocytes were centrifuged at 1800 rpm for 15 min and obtained cell pellet was resuspended in RBC lysis buffer (BD biosciences, USA). Following incubation for 5 min at 37 °C, splenocytes were centrifuged at 1800 rpm for 15 min and collected cell pellet resuspended in complete RPMI containing 10% FBS (Cat no. 0010, Hyclone, USA). Splenocytes (0.5 × 10^6^ cells) were cultured either with empty device, ECB-BDNF device, or lipopolysaccharides (LPS) (10 µg/mL, Sigma-Aldrich, USA) in 96-well flat bottom plate for 48 h and blocked for protein transport using golgi stop containing monensin during last 4 h of incubation (BD biosciences, USA). Splenocytes were collected and processed for flow cytometry analysis by surface staining with brilliant violet 510 conjugated anti-CD3 and APC-Cy7 conjugated anti-B220 antibody (BioLegend, USA). Surface-stained cells were fixed and permeabilized using Foxp3 transcription factor fixation/permeabilization concentrate and diluent (eBioscience, USA) for 30 min at 4 °C followed by intracellular staining at 4 °C for 30 min against TNF-α using brilliant violet 711 conjugated anti-TNF-α monoclonal antibody (BioLegend, USA). Samples were analyzed on a BD LSR II flow cytometer and data was analyzed with FlowJo (version 10.8.1) software.

To analyze the immune cells which were adhered to ECB devices during its explantation after study completion as described in "ELISA" Section, the ECB devices were washed thoroughly with warm PBS and the cells were collected. Samples were pooled resulting in one set of WT and *App*^*NL−G−F*^ samples each time-point, since cells in individual samples were not enough for analysis. All the adhered cells were only surface stained for various cell determinants including CD45 (pan-leukocytes marker), CD3 (pan-T cell marker), B220 (pan-B cell marker), CD11b (microglia/macrophage marker), CD11c (DC, Dendritic cells marker), and NKp46 (NK, Natural killer cell marker), respectively. Samples were analyzed on a BD LSR II flow cytometer and data was analyzed with FlowJo (version 10.8.1) software.

### ELISA

BDNF release from the ECB-BDNF devices were measured prior to their implantation into mice brain. ECB-BDNF devices were maintained in 1 mL of fresh HE-SFM in 12-well plates (Corning, New York, NY, USA) for 4 h at 37 °C and 5% CO_2_, and 500 µL of the supernatant was collected and saved for future BDNF analysis at − 80 °C freezer. Post-explantation of the ECB-BDNF devices from the brains of different cohort of mice as described in method "Study design for ECB implantation" Section, the devices were thoroughly washed with warm PBS to wash away any cells sticking onto the devices. The collected cells were pooled within each group (WT or *App*^*NL−G−F*^*)* and analyzed by flow cytometry as described in method "Flow cytometry" Section. The devices were then kept in fresh 1 mL HE-SFM for 4 h at 37 °C and 5% CO_2_, within a period of 24 h post-explantation, and 500 µL supernatant was collected for future analysis as described below.

Total BDNF content in ECB-BDNF device supernatants was measured using the DuoSet human/mouse BDNF ELISA kit (DY248, R&D Systems) according to the manufacturer’s protocol, with minor modification. Briefly, 384-well plates (464,718, Nunc MaxiSorp) were coated with 50 µL of 2 µg/mL capture antibody prepared in carbonate buffer, pH 9.8, and incubated overnight at 4 °C. Plates were then washed once with 100 µL of tris-buffered saline (TBS) followed by the addition of 50 µL of 5% BSA prepared in carbonate buffer to block unspecific epitopes and incubated for 1 h at room temperature. Plates were then washed 3 × 5 min each using 100 µL of TBST (TBS + 0.05% Tween20) and 50 µL of individual samples were added to their respective wells. Purified BDNF protein prepared in reagent diluent (1% BSA in PBS, 0.01% sodium azide, 0.22 μm filtered, pH 7.4) was used as standard reference wells (S1 = 4 ng/mL, serially diluted until S10). After overnight incubation, plates were again washed 3 × 5 min each using 100 µL of TBST followed by the addition of 50 µL of 50 ng/mL detection antibody prepared in reagent diluent and further incubated for 3 h at room temperature. Plates were then washed 3 × 5 min using TBST and incubated with 50 µL streptavidin conjugated alkaline phosphatase diluted in reagent diluent (1:10,000; #11,093,266,910, Roche Diagnostics) for 2 h at room temperature. Plates were then washed 2 × 5 min with TBST and 1 × 5 min using diethanolamine (DEA; 1.0 M, pH 9.8). Alkaline phosphatase substrate (p-Nitrophenyl-Na_2_-6H_2_O, 50 µL/well, Sigma-Aldrich) was then added, and absorbance was kinetically read in a spectrophotometer (Infinite M1000, Tecan) at 405 nm for 1 h using 5 min interval.

### Immunofluorescence

Paraffin-embedded brain tissues were sectioned into 4-mm-thick sections. The tissues were deparaffinized by washing in xylene and in decreasing (99–70%) concentrations of ethanol. For antigen retrieval, slides were pressure boiled in citrate buffer solution (0.1 M citric acid and 0.1 M sodium citrate) at 110 °C for 5 min and then washed with tap water followed by PBS-Tween 0.05% for 5 min each. Sections were then incubated with NGS (normal goat serum, Vector Laboratories, USA) for 30 min at room temperature. The brain sections were then incubated with the primary antibody at 4 °C, overnight (see Supplementary Table 1). On the second day, the positive staining signal was amplified by using TSA Fluorescence System kit (NEL701A001KT, Akoya Biosciences, USA). Briefly, sections were incubated with biotinylated anti-mouse or anti-rabbit antibodies (Vector Laboratories; UK) 1:200 in Tris-NaCl-blocking buffer (TNB) or NGS for 2 h at room temperature and then incubated with horseradish peroxidase (HRP)-conjugated to streptavidin (PerkinElmer; USA) 1:100 in TNB buffer or NGS for 30 min. For signal amplification, samples were incubated for 10 min in tyramide (PerkinElmer; USA) 1:50 in Amplification Reagent. Samples were incubated for 15 min with slow agitation with Hoechst solution (1:500 in PBS-T) for nuclei staining wherever needed, followed by mounting with PermaFluor Aqueous Mounting Medium (ThermoScientific, USA) and kept for drying overnight. Between each incubation step, samples were washed 3 × in PBS-T for 5 min with slow agitation. Images were acquired with digital Camera (Nikon D5-Qi2) connected to a Nikon fluorescence microscope (Nikon Eclipse E800) with a Plan-Apochromate 2 × , 4 × , 10 × , and 20 × objectives. The sections were then visualized with Nikon Eclipse E800 confocal microscope and imaged with Nikon DS-Qi2 camera for further analysis on ImageJ software (National Institutes of Health, Bethesda, USA).

### Quantification of immunohistochemical staining

Staining for Aβ plaque deposition along with BDNF, CD45, GFAP, fibroblast, and Iba1 immunoreactivity were quantified using ImageJ. During imaging acquisition, exposure time and numeric gain were kept constant between slides to avoid potential technical artefacts. Images were first converted to 8-bit gray scale and binary thresholder to highlight a positive staining. For quantification of immunoreactivity around ECBs, two circular regions of interest were drawn around the center of the ECBs—a proximity region with a diameter of 1000 pixels and a distal region with a diameter of 2500 pixels. For the total cell count of microglia and astrocytes, we counted Iba1 and GFAP stained cells, respectively. BDNF protein (248-BDB-010, RnD systems) was used to block anti-BDNF antibody prior to staining as a control to investigate the specificity of the BDNF antibody.

### Statistical analysis

All statistical comparisons were performed using Prism 8 (GraphPad software Inc., CA, USA). Data were analyzed by two-way ANOVA with Bonferroni correction for multiple comparisons. All other data were analyzed by multiple *t*-tests or two-tailed Student’s *t*-test. Error propagation was used to account for variation between sections from the same mouse for immunostaining experiments. Variability of the estimates was reported as the standard error of the mean (SEM); *p* < 0.05 was considered as statistically significant.

## Results

### *BDNF gene expression is downregulated in hippocampus of App*^*NL−G−F*^* mice*

**Fig. 1:**
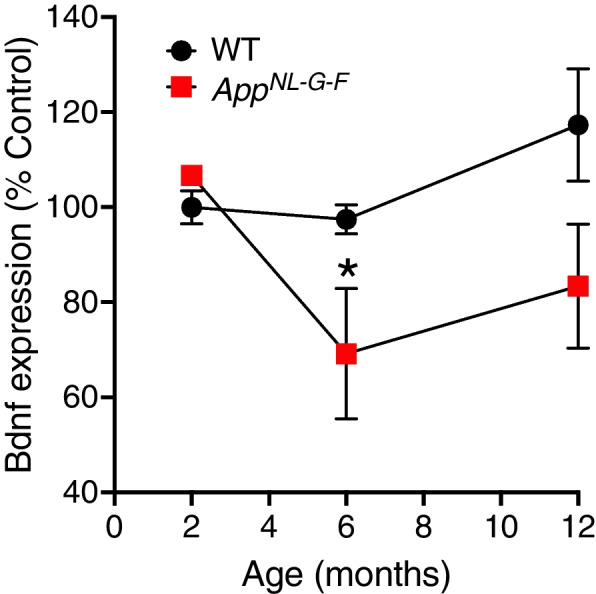
BDNF gene expression is downregulated in *App*^*NL−G−F*^ mice. Hippocampal BDNF mRNA levels are significantly lower (*p* < 0.05) in *App*^*NL−G−F*^ mice at 6 months of age compared to age-matched control WT mice (*n* = 3). Data across the different time points were analyzed by two-way ANOVA with Bonferroni correction for multiple comparisons

To investigate the levels of BDNF gene expression, we analyzed a previously generated data set (Nilsson personal communication) in which we have performed transcriptome analysis of hippocampus of *App*^*NL−G−F*^ mice at 2, 6, and 12 months of age. This revealed a significant downregulation of BDNF mRNA levels at 6 months of age compared to WT controls (Fig. 1). At this age, the *App*^*NL−G−F*^ mice start to have memory impairment [36].

### ECB-BDNF releasing devices exhibited low immunogenicity ex vivo

**Fig. 2:**
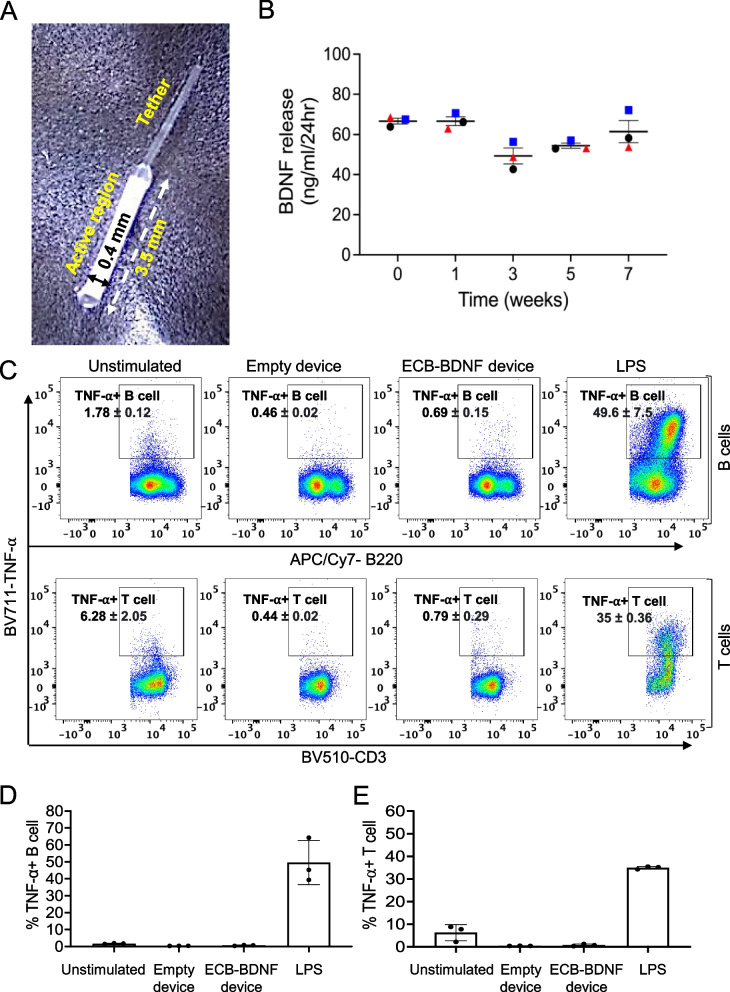
Design of miniaturized ECB device, in vitro BDNF release quantification and ex vivo immune response. **A** Representative picture of a miniaturized ECB device. **B** BDNF release (ng/mL/24 h) from three independent ECB-BDNF devices (black, red, and blue symbols) were monitored for 7 weeks, where BDNF levels were measured by ELISA. **C** Representative dot plots and **D**, **E** bar diagram, depicts the TNF-⍺ positivity among the B and T cells in splenocytes isolated from WT mice which were incubated with different ECB devices for 48 h and examined by flow cytometry (*n* = 3). Data are represented as mean ± S.E.M. and analyzed by unpaired Student’s *t*-test

Mouse-sized ECB-BDNF releasing devices (Fig. 2A) stably released BDNF over several weeks (measured up to 7 weeks) in vitro (Fig. 2B). Furthermore, to elucidate the feasibility of using ECB devices for in vivo implantation in WT and *App*^*NL−G−F*^ mice, we first evaluated the immunogenicity of the ECB devices by co-culturing empty and ECB-BDNF devices with primary WT mouse splenocytes ex vivo*,* using unstimulated primary mouse splenocytes as control (Fig. 2C). Following 48 h of incubation, there was no increase in TNF-α-positive B cells in response to neither the empty device (0.46%) nor the ECB-BDNF device (0.69%), compared to unstimulated splenic B cells (1.78%). Similarly, we observed 0.44% and 0.79% of total splenic T cells to be expressing TNF- α following exposure to empty or ECB-BDNF device respectively, compared to 6.28% in unstimulated group. In contrast, LPS treatment induced 49.6% B cells and 35% T cells to express increased TNF- α levels, respectively. Overall data were represented as bar plots (Fig. 2D and E), demonstrating that neither the ECB biomaterial nor the antigens shed by the ECB-BDNF devices could significantly activate mouse primary splenocytes ex vivo.

### ECB-BDNF devices were well tolerated and increased BDNF levels in the brain

**Fig. 3:**
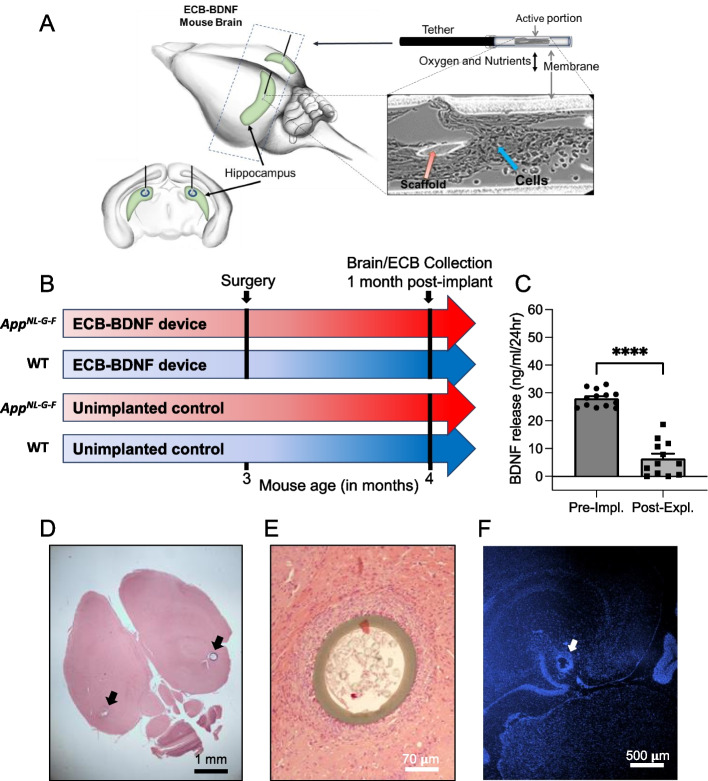
Schematic representation of the ECB implantation into mouse hippocampus, ECB brain parenchymal localization and BDNF release pre- and post-implantation. **A** Schematic representation of the bilateral implantation of ECB device in mouse hippocampi. **B** Representation of the Study outline using cohort 1 mice. **C** Level of BDNF release from the ECB-BDNF devices during pre-implantation (pre-impl.) and post-explantation (post-expl.). Data are represented as mean ± S.E.M. and analyzed by unpaired Student’s *t*-test. **D** Representative ECB localization in mouse brain stained with H&E, **E** its integration to brain parenchyma stained with H&E, and **F** distribution of cells around the ECB devices as stained with Hoechst

To evaluate the feasibility and tolerability of using the ECB-BDNF devices in vivo, 3-month-old WT and *App*^*NL−G−F*^ mice with progressing AD pathology were implanted bilaterally into hippocampus for 1 month (cohort 1) (Fig. 3A, B). The surgical procedure did not affect the health of the mice post-operatively, indicated by 100% survival and normal body weight (Supplementary Figure 1). The ECB-BDNF devices after 1-month post-explantation continued releasing BDNF but at significantly reduced levels compared with pre-implantation levels. The level of BDNF released by the devices were similar when explanted from WT or *App*^*NL−G−F*^ mice, thus the mean data was represented as bar plots (Fig. 3C). The correct positioning of the implants was confirmed by histological analysis. The implants were found to be integrated in the brain parenchyma with increased cell density in the immediate proximity of the implants (Fig. 3D-F).

**Fig. 4:**
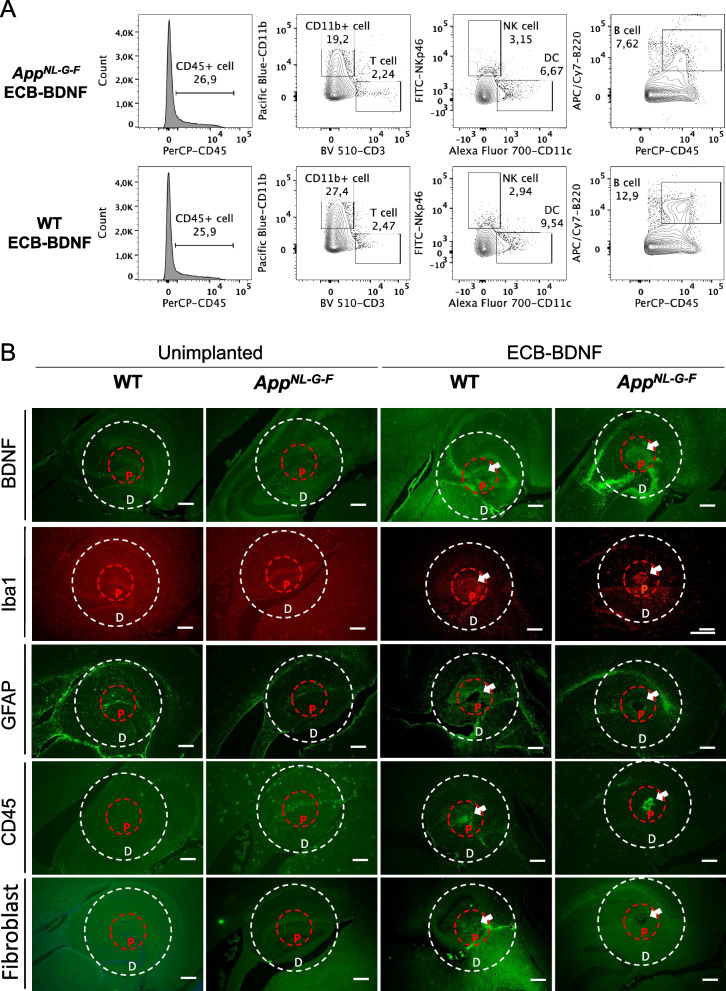
Flow cytometry and immunofluorescence analysis revealed considerable differences in the inflammatory response comparing WT and *App*^*NL−G−F*^ mice. **A** ECB-adhering cells at post-explantation were collected, pooled, and surface stained for various immune cell markers and analyzed by flow cytometry. Pan-leukocyte marker CD45 was used to select immune cells and was sub-gated to identify specific cell types including T-, natural killer (NK)-, B-, dendritic (DC)-, and microglia-macrophage (CD11b) cells, respectively. **B** Representative immunohistochemical images showing BDNF status, microglia (Iba1), astrocytes (GFAP), leukocyte (CD45), and fibroblasts in the proximity (P) and distal (D) area surrounding the ECB-BDNF devices after 1 month of treatment (*n* = 2–4). Scale bar 300 μm

Flow cytometry analysis of the cells adhering to the implants 1-month post implantation revealed major differences in the population of immune cell comparing WT and *App*^*NL−G−F*^ mice (Fig. 4A). Although the overall population of immune cells (CD45^+^, pan-leukocyte marker) were unaltered, *App*^*NL−G−F*^ mice displayed considerably less accumulation of microglia/macrophage (CD45^+^CD11b^+^), dendritic cells (DC, CD45^+^CD11c^+^), and B cells (CD45^+^B220^+^), respectively. We did not observe major changes in accumulation of T-cell (CD45^+^CD3^+^) and natural killer cells (NK, CD45^+^NKp46^+^).

**Fig. 5:**
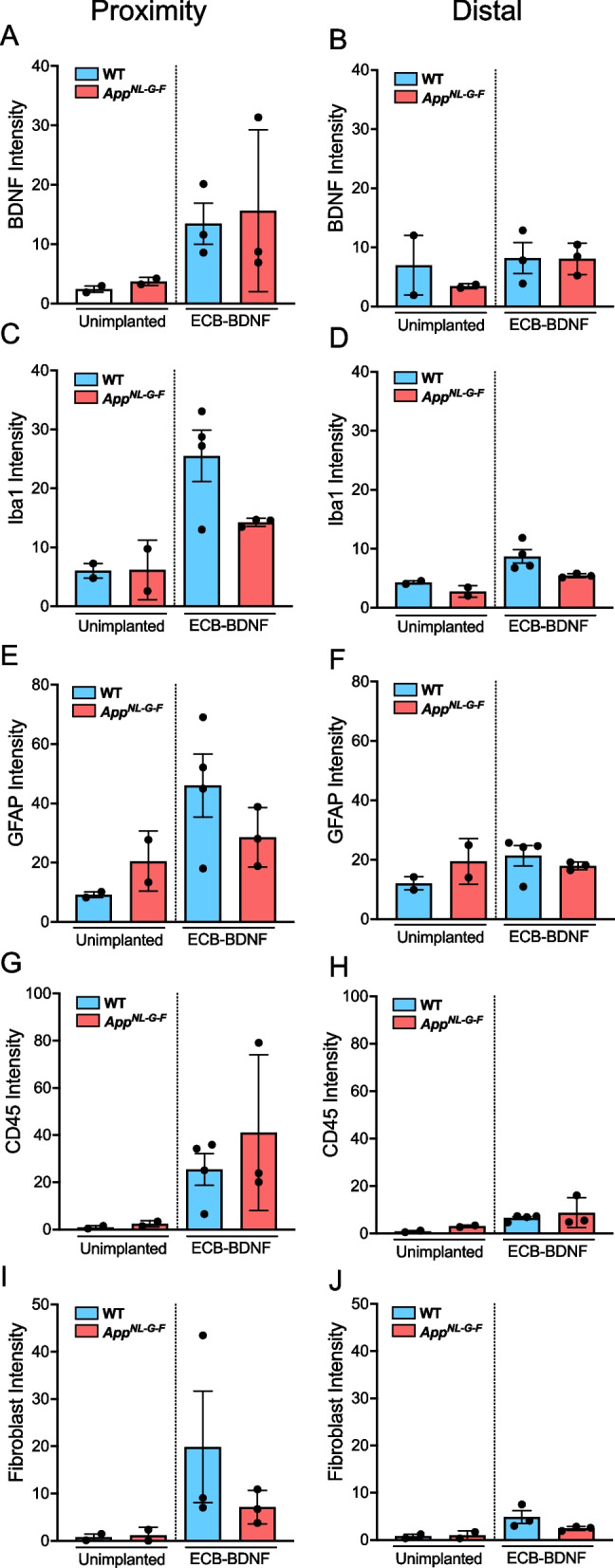
Immunofluorescence analysis revealed response to the implanted ECBs at 1-month post-surgery. Immunofluorescence intensity analysis of **A**,** B** BDNF release, **C**, **D** microglia (Iba1), **E**, **F** astrocytes (GFAP), **G**, **H** leukocytes (CD45), and **I**, **J** fibroblasts; in the proximity and distal area surrounding the ECB-BDNF devices in control (un-implanted) and ECB-BDNF implanted mice in cohort 1 (*n* = 2–4). Statistical comparison between groups were not applied due to the limited number of animals in control groups. Data represented as mean ± S.E.M

Positive BDNF staining was found only in the ECB-BDNF implant proximity area in both WT and *App*^*NL−G−F*^ mice (specificity of anti-BDNF antibody is shown in Supplementary Fig. 2A), where both implanted groups had higher staining as compared to the unimplanted control mice from respective genotypes (Figs. 4B and 5A, B). Immunostaining for cells involved in inflammatory response towards the implanted ECB-BDNF devices revealed a trend towards increased microgliosis (Iba1) but not astrocytosis in the area surrounding the implants, as determined by the intensity of the staining and the number of microglia cells as compared to the un-implanted mice (Figs. 4B, 5C-F, Supplementary Fig S3A-D, Supplementary Tables 2 and 3). There was a mild trend towards less inflammatory response in implanted *App*^*NL−G−F*^ mice compared to implanted WT. Quantitative leukocyte staining revealed a trend towards increased CD45 staining and fibroblasts in both proximal and distal brain regions in the implanted mice brain, compared to the unimplanted mice brain tissues (Fig. 5G–J). No positive staining for IgG, a marker of altered BBB permeability, was observed (Supplementary Fig. 2B). These experiments were performed on a smaller number of animals; thus, statistical analysis with multiple comparison was not applicable (raw data for mean ± S.E.M are provided in Supplementary Tables 2 and 3).

### AppNL-G-F mice displayed reduced inflammation and plaque deposition around ECB-BDNF implants, lowered anxiety-related behavior, and improved spontaneous Y-maze alternation

**Fig. 6:**
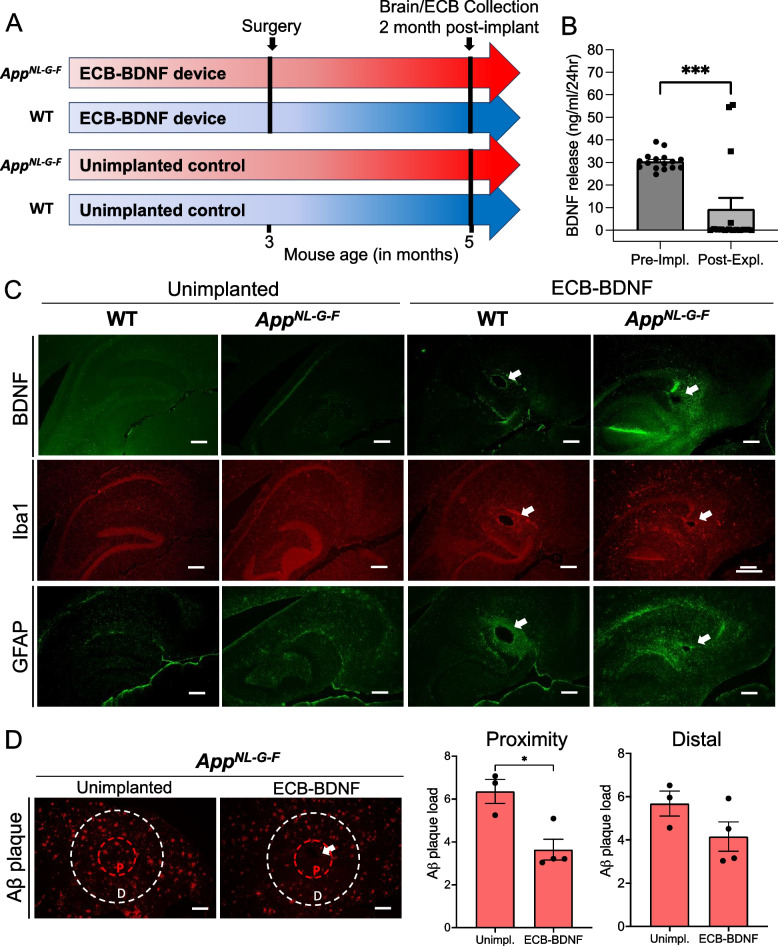
Two months of treatment with ECB-BDNF in *App*^*NL−G−F*^ mice lowered Aβ plaque deposition around implant proximity. **A** Schematic representation of ECB implantation timeline in cohort 2. **B** BDNF released from the ECB-BDNF devices at pre-implantation (pre-impl.) and after explantation (post-expl.) in cohort 2. **C** Representative immunofluorescence staining of BDNF levels, microglia (Iba1), and astrocytes (GFAP) in mouse brain slices from non-implanted and ECB-BDNF implanted mice (*n* = 2). **D** Analysis of Aβ plaque deposition staining in the proximity and distal areas surrounding ECB-BDNF devices (*n* = 4). Scale bar 300 μm. Data are represented as mean ± S.E.M. Data were analyzed by unpaired Student’s *t*-test

Utilizing cohort 2 animals, the effect of ECB-BDNF devices were evaluated for 2 months in *App*^*NL−G−F*^ and WT mice (Fig. 6A). The ECB-BDNF devices released significantly reduced BDNF levels, almost 60% reduction at 2-month post-explantation, as compared to pre-implantation levels (Fig. 6B). Positive GFAP and Iba1 staining was observed as well as a BDNF-positive staining in the proximity of the implanted area (Fig. 6C), compared to the levels observed in unimplanted control tissues. Among the implanted groups, increased immunoreactivities towards BDNF, GFAP, and Iba1 were observed in the proximity of the implanted area of *App*^*NL−G−F*^ mice compared with the implanted WT mice (Fig. 6C). Interestingly, Aβ plaque deposition in the proximity of the implants was significantly reduced in the 2-month ECB-BDNF implanted *App*^*NL−G−F*^ mice compared with unimplanted *App*^*NL−G−F*^ (Fig. 6D).

**Fig. 7:**
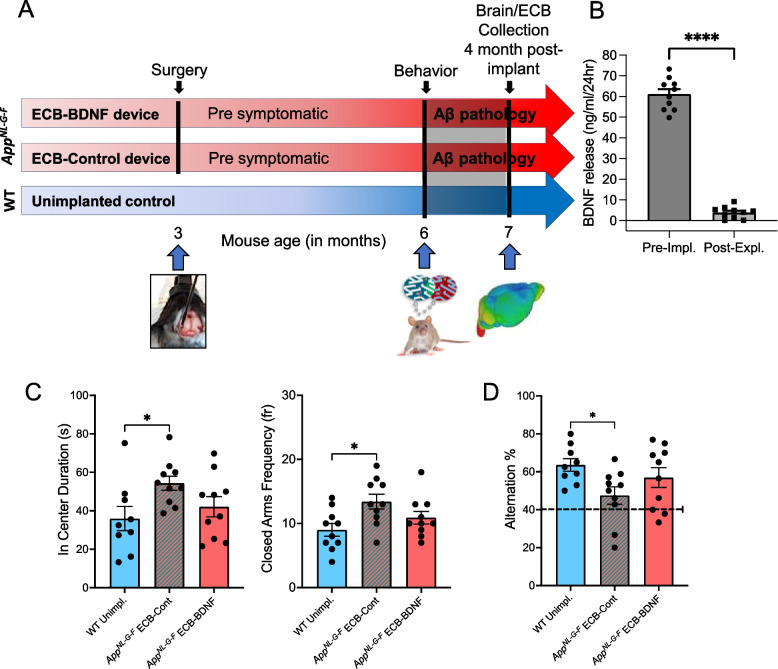
BDNF treatment effects on anxiety and spatial learning behavior in the App^*NL−G−F*^ mice. **A** Schematic representation of the study design in cohort 3. **B** BDNF release of ECBs at pre-implantation (pre-impl.) and after explantation (post-expl.) in cohort 3. **C** Three-month BDNF treatment effect on anxiety in mice using elevated plus maze (EPM), and **D** the spontaneous Y maze alternation in *App*^*NL−G−F*^ mice (*n* = 9–10). Data are represented as mean ± S.E.M. Data were analyzed by one-way ANOVA with Tukey’s multiple comparison test

This prompted us to evaluate the efficacy of treatment with BDNF-releasing devices on behavioral parameters like anxiety-, learning-, and memory-related behavior which was performed using a separate cohort of animals (cohort 3; Fig. 7A). Although the release of BDNF post-explantation was reduced by 90% as compared to pre-implantation levels at 4 months (Fig. 7B), various changes in behavior were observed among the ECB-BDNF implanted animals. The *App*^*NL−G−F*^ mice implanted with the ECB-Control devices spent significantly increased time in the center and visited the closed arms with a significantly higher frequency than the WT unimplanted mice in the elevated plus maze test, confirming an increased anxiety like behavior in the *App*^*NL−G−F*^ mice (Fig. 7C). On the other hand, the performance of the mice implanted with ECB-BDNF devices were not significantly different as compared to the WT unimplanted mice indicating a positive effect of BDNF in reducing anxiety-related behavior (Fig. 7C). A similar positive effect was observed in the Y-maze test showing that the ECB-BDNF group performed like the unimplanted WT mice, whereas ECB-Control group showed a significant cognitive deficit in alternation compared with the unimplanted WT mice (Fig. 7D). However, the ECB-BDNF group were not significantly different from the ECB-Control group, indicating an intermediate phenotype.

**Fig. 8:**
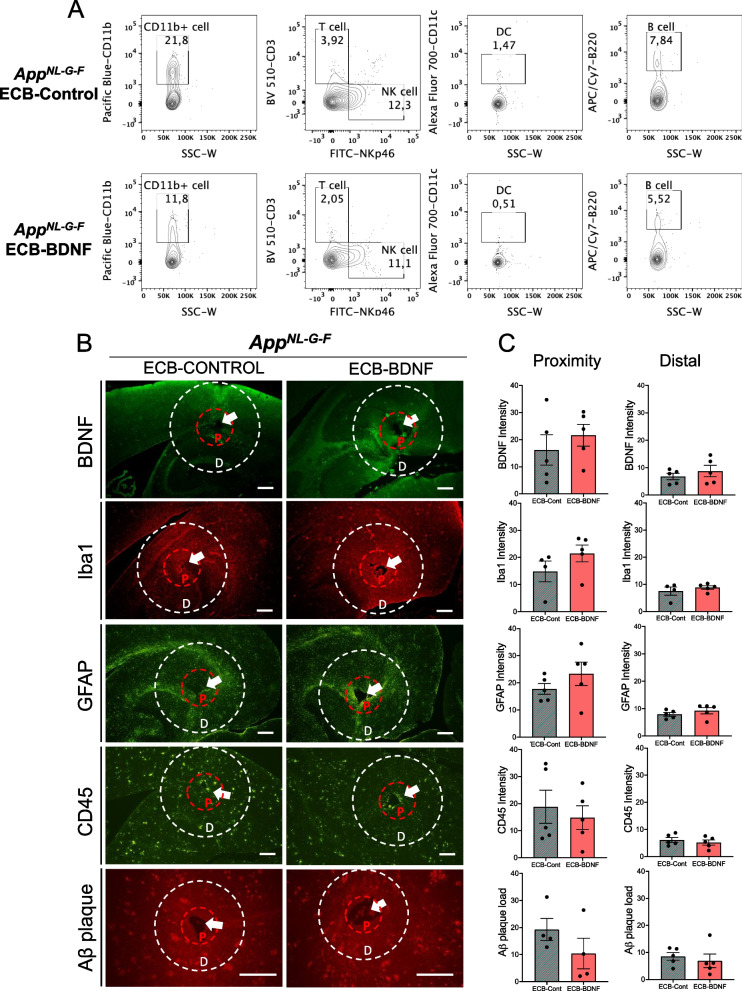
Flow cytometry and immunofluorescence analysis of the neuroinflammatory response to ECB implants at 4 months. **A** ECB-adhering cells at post-explantation were collected, pooled, and surface stained for various immune cell markers and analyzed by flow cytometry. Pan-leukocyte marker CD45 was used to select immune cells and was sub-gated to identify specific cell types including T-, natural killer (NK)-, B-, dendritic (DC)-, and microglia-macrophage (CD11b) cells, respectively. **B** Representative immunofluorescence images of BDNF levels, microglia (Iba1), astrocytes (GFAP), leukocytes (CD45), and Aβ plaques staining. Scale bar 300 μm. **C** Specific staining were analyzed in the proximity and in the distal area surrounding the ECB devices and presented as bar-plots (*n* = 4–5). Data are represented as mean ± S.E.M. Data were analyzed by unpaired Student’s *t*-test

In addition, flow cytometry analysis revealed a 50% reduction in CD11b^+^ cells adhering to ECB-BDNF devices (11.8%) compared with ECB-Control implants (21.8%) (Fig. 8A). Considerable reduction was also observed in T-, dendritic, and B cell populations in the ECB-BDNF group, respectively. Using immunohistochemistry on tissue sections, we could not find any significant differences in the intensity of Iba1, GFAP, CD45, and BDNF in *App*^*NL−G−F*^ mice implanted with ECB-BDNF as compared to *App*^*NL−G−F*^ mice implanted with ECB-Control (Fig. 8B, C). Total number of astrocytes (GFAP) and microglia (Iba1) cells in the surrounding proximity and distal area of ECBs implanted *App*^*NL−G−F*^ mice were also analyzed but no significant differences were found (Supplementary Fig. 3E-H and Supplementary Table 3) indicating that the trend towards increased inflammatory response observed in the early stage of implantation (1–2-month post implantation) was recovered. A non-significant trend of reduced Aβ plaque deposition in the proximity of the implanted area in the ECB-BDNF group as compared to ECB-Control group was observed (*p* = 0.09, Fig. 8B, C).

## Discussion

In this study, we explored the feasibility and therapeutic potential of local intracerebral encapsulated biodelivery of BDNF into the brain of *App*^*NL−G−F*^ AD mice. We show the feasibility and tolerability of the miniaturized ECB devices adapted for mouse brains which open opportunities to study the intracerebral delivery of drugs safely and precisely, in pre-clinical mouse models. Interestingly, reduced BDNF gene expression in hippocampal tissues of *App*^*NL−G−F*^ mice overlaps with the same age (6 months) when the first sign of memory impairment appears [36, 42]. Importantly, our findings align with the results obtained in post-mortem AD brains, where lower levels of BDNF have been observed in the hippocampus and the temporal and frontal cortices [30]. In addition, serum BDNF levels have been found to be reduced in subjects with mild cognitive impairment (MCI) and AD and correlated with the severity of memory impairment [33]. We found here that intracerebral hippocampal delivery of BDNF marginally reduces the extent of memory alteration in *App*^*NL−G−F*^ mice.

Several pre-clinical studies have been performed to evaluate the therapeutic potential of BDNF [43]. However, neurotrophic factors, including BDNF, do not cross the BBB [34]. Therefore, developing a controlled drug delivery system to increase BDNF protein levels in specific brain regions may provide the opportunity to explore its therapeutic potential. Taking this into consideration, several CNS delivery strategies have been explored, such as direct injection into the brain, expression by viral vectors, brain infusion pump, trans-nasal drug delivery, and transient disruption of BBB by focused ultrasound [2, 44, 45]. Relevant studies in the field have been conducted by Tuszynski and his colleagues, using lentivirus-expressing BDNF injected in the entorhinal cortex in both mice and primates which reversed atrophy and improved cognitive impairment [46–48]. Surprisingly, their efforts to deliver AAV-NGF in previous clinical trials in AD patients received setbacks from improper stereotactic injections and inefficient target-engagement [49]. To solve drug delivery problems across BBB and to evaluate the clinical promise of different neuroprotective factors, new innovative technologies or alternative methods of drug delivery are required [2]. One of the promising drug delivery methods developed to achieve these goals is the ECB platform [3]. This technique is based on genetically modified cells that produce a therapeutic protein encapsulated within a semi-permeable membrane. The ECB technique possesses the qualities of targeted in-situ delivery, long-term release, with a retrievable biologically safe method.

ECB engineering led to the first successful open-label human phase 1 clinical trial using ECB-NGF devices in 6 AD patients by our group [3, 4]. This study showed that ECB-NGF therapy in AD patients is feasible, safe, and well tolerated for up to 1 year (experimental endpoint of the study). Improvement in cognition, electroencephalogram (EEG), and nicotinic receptor binding were reported, along with optimum target engagement and improved cholinergic markers in cerebrospinal fluid (CSF) in three patients [4, 6, 50, 51]. It is worthwhile to mention that among the 6 patients receiving bilateral 1st generation ECB-NGF devices, half of them showed reduced MMSE decline and brain atrophy as compared to a general reference group [6, 51]. An improved version (second generation) of the ECB-NGF devices was developed which released ten times more NGF and was utilized in a follow-up dose escalation study in four AD patients for 6 months, confirming the observations of beneficial effects of first trial but without halting the decline in MMSE [5, 50, 52]. The data taken together indicate positive effects of the NGF application via ECB platform. Among the patients in this trial, individuals who showed cognitive improvement during the active administration period proved to be those with superior implant function, as measured by NGF release and histologically at explantation upon study completion [4, 5, 51, 53].

We have reported previously that the levels of neurotrophin released from the ECB devices were altered when the devices were removed from the brain after treatment period [3], which could explain the variation in clinical response. One plausible explanation could be that the AD associated pathology and inflammatory factors had affected the encapsulated cells within the ECB devices. In the present study, the use of genetically identical pre-clinical models maintained within controlled surroundings allowed us to address these issues by reducing several of the variabilities associated with the clinical trials. Interestingly, we also observed reduced BDNF release from the devices over time indicating that factors related to the surgical implantation, associated inflammation, and the response of BDNF releasing cells towards these factors should be studied further to optimize ECB-mediated long-term drug release in the brain tissue. The ECB-BDNF devices which were maintained in vitro remained viable and showed stable BDNF release over almost 2 months. Whereas the ECB-BDNF devices implanted to the mouse brain in vivo exhibited reduced BDNF release as observed at 1-, 2-, and 4-month post-implantation. This indicates that brain associated factors may play a considerable role in modulating drug release from the ECB devices, either by affecting the BDNF production per se, cell survival, or transport over the semi-permeable membrane. Moreover, we observed a similar range of post-explanation BDNF release from both the WT and the *App*^*NL−G−F*^ mice, indicating that AD pathology in *App*^*NL−G−F*^ mice did not have any specific effect in reducing BDNF release. We had previously shown that Aβ alone marginally affects physiological parameters of encapsulated cells, while glial cells have comparable or higher potency to impair ECB-mediated drug release or the cellular activities [54, 55]. All these aspects should be taken into consideration when planning future work utilizing ECB mediated drug delivery.

To evaluate the immunogenic potential of the ECB-BDNF devices engineered to release human BDNF, we examined the mouse (host) adaptive immune response against the devices. We utilized ex vivo co-culture platform capable of delineating host adaptive immune response against encapsulated cells [56]. Interestingly, we found that neither the biomaterial nor the antigens, including human BDNF or other antigens shed by the encapsulated human cells in the ECB device, activated the mouse immune cells to release cytokines. We further explored the in vivo immunogenicity of ECB devices in the *App*^*NL−G−F*^ mice and studied immune cells around the device in tissue sections as well as the types of immune cells attached to the ECB-BDNF devices. We found that the ECB-BDNF devices were well-tolerated in both WT and *App*^*NL−G−F*^ mice, with 100% survival rate, and displayed less immunogenicity in the *App*^*NL−G−F*^ mice.

Most importantly, after 2 months of BDNF delivery in mice cohort 2, a reduction in Aβ plaque deposition was observed in the areas adjacent to the devices. Previously, an effect on Aβ plaque load was reported after intranasally delivered NGF in 5xFAD mice, but not for BDNF [57]. In our study, we used a different delivery system which permits BDNF to be released directly in the target area. A direct effect of BDNF on the amyloid cascade is not yet reported in the literature, so it is possible that BDNF may affect Aβ production and aggregation indirectly [58, 59]. Indeed, in situ hybridization has revealed BDNF mRNA signals associated with Aβ plaques [59]. On the other hand, we speculate that the observed effect could be mediated through a dual interaction of BDNF with neurons and microglia. It is known that BDNF improves cell survival and synaptic function [28] and inhibits microglial activation [23]. In this regard, we have observed a 50% decrease in microglia sticking to the implants, as assessed by the number of CD11b positive cells after four months of implantation in cohort 3 of mice.

The positive effect of the hippocampal release of BDNF was also observed as a tendency towards an improvement in anxiolytic-like behavior and spatial memory-like performance in mice cohort 3. An antidepressant-like behavioral effect of BDNF has previously been reported in mice [60]. Taken together, our data support that BDNF treatment may have the potential to ameliorate neuropsychiatric symptoms in AD, but further studies are needed. Even though BDNF production and release from ECBs kept in vitro exhibited a stable dosage, post-implantation analysis of the ECB-BDNF device revealed a gradual decline in the BDNF release after 1-, 2-, and 4- months post-implantation. Since the BDNF release from the ECBs was reduced after 4 months post-implantation, this needs to be stabilized to improve the therapeutic feasibility. Overall, the study provides a strong basis for the use of the ECB-BDNF platform as a potential therapeutic approach to treat AD and showcases the applicability of the miniaturized ECB platform for drug delivery in pre-clinical studies.

### Limitations of the study

Since there is a growing interest in the AD field in developing anti-Aβ therapies, we decided to evaluate the therapeutic potential of ECB-BDNF in a model which closely resembles Aβ pathology and cognitive impairment (*App*^*NL−G−F*^). This mouse model allows us to study the effect of Aβ on cognition, without any interference from other aspects (tau phosphorylation and neuronal death). This also allows us to evaluate the therapeutic potential of BDNF in restoring cognitive behavior, especially while counteracting Aβ-specific pathology.

The number of mice used for cohort 1 and cohort 2 to evaluate the survival and tolerability of the implants was kept low for feasibility and ethical purposes. Though this excluded a comparison between the individual groups, an analysis of treatment effect comparing treated and non-treated groups across genotypes could be performed. On the other hand, the number of mice used for cohort 3, which were assessed by behavioral measurements, were substantially higher to retain power in the analysis. For this part of the study, a control group of *App*^*NL−G−F*^ mice could have been added to address whether the implants themselves specifically affected the *App*^*NL−G−F*^ mice including potential immune response after 4 months of treatment. However, a lack of immune response towards the ECB devices were shown in cohort 1 and 2, which ruled out the involvement of implant-induced immune activity against the ECB devices.

A limitation that may have resulted in unwarranted variation could be related to the size of the implant and the brain of the mice. Even using the miniaturized version of ECB implants, the disproportionality between the relative size of the human brain to the mouse brain is very difficult, if not impossible, to cover. Thereby, larger animal models could be more appropriate for future studies, e.g., rats instead of mice model of AD. The reduced levels of BDNF production from the implants need to be addressed for future application.

## Conclusions

We show here that the ECB platform is a viable method to safely deliver proteinaceous drugs in mouse brain hippocampus, although the BDNF delivery dosage needs to be stabilized for long-term treatments. Furthermore, this study demonstrated that BDNF delivery in the hippocampal region may alleviate AD-associated pathology in the proximity of the implantation site. Although more studies are needed to validate our findings in the AD brain, this study sheds light on the potential of BDNF therapy for controlling glial activation and inflammation-associated neurodegenerative processes. These data provide support for the feasibility of the miniaturized ECB devices as an optimal drug delivery platform, warranting further development of this approach as a potential treatment for AD.

### Supplementary Information

**Additional file 1: Figure S1.** Mouse body weight after bilateral ECB implantation in hippocampus. **Figure S2.** Specificity of BDNF antibody and immunofluorescence staining for IgG, CD45 and fibroblasts. **Figure S3.** Immunofluorescence analysis of microglia and astrocyte cells in the proximity and distal area surrounding the implanted ECBs at one-month and four-month post-surgery.

**Additional file 2: Supplementary Table 1. **List of the antibodies.

**Additional file 3: Supplementary Table 2.** Data for Fig. 5.

**Additional file 4: Supplementary Table 3**. Data for Supplementary Fig. 3.

## Data Availability

The datasets used and/or analyzed during the current study are available from the corresponding author on reasonable request.

## Abbreviations

Aβ: Amyloid-βeta
AD: Alzheimer’s disease
APP: Amyloid precursor protein
ARPE-19: Spontaneously arising retinal pigment epithelia cell line
App^NL-G-F^: APP-knock-in mice
ANOVA: Analysis of variance
BBB: Blood–brain barrier
BDNF: Brain-derived neurotrophic factor
CNS: Central nervous system
CSF: Cerebrospinal fluid
DEA: Diethanolamine
DMEM/F12: Dulbecco’s modified Eagle medium/nutrient mixture F-12
ECB: Encapsulated cell biodelivery
EEG: Electroencephalogram
EPM: Elevated plus maze
ELISA: Enzyme-linked immunosorbent assay
FBS: Fetal bovine serum
GFAP: Glial fibrillary acidic protein
HE-SFM: Human endothelial serum-free media
HRP: Horseradish peroxidase
Iba1: Ionized calcium binding adaptor molecule 1
LPS: Lipopolysaccharides
MCI: Mild cognitive impairment
NMDA: N-methyl-D-aspartate
NGF: Nerve growth factor
NGS: Normal goat serum
RPMI: Roswell Park Memorial Institute 1640 medium
TBS: Tris-buffered saline
TBST: TBS + 0.05% Tween20
TNB: Tris-NaCl-blocking buffer
TNF-a: Tumor necrosis factor α
TrkB: Tropomyosin receptor kinase B receptor
WT: Wild type
