# Supplementary material for: Feasibility and therapeutical potential of local intracerebral encapsulated cell biodelivery of BDNF to AppNL−G−F knock-in Alzheimer mice
Source: Alzheimers Res Ther. 2023 Aug 18;15:137. doi: 10.1186/s13195-023-01282-x (PMC10436657; doi:10.1186/s13195-023-01282-x)
Supplement: Supplementary file 1 — Additional file 1: Figure S1. Mouse body weight after bilateral ECB implantation in hippocampus. Figure S2. Specificity of BDNF antibody and immunofluorescence staining for IgG, CD45 and fibroblasts. Figure S3. Immunofluorescence analysis of microglia and astrocyte cells in the proximity and distal area surrounding the implanted ECBs at one-month and four-month post-surgery. [file 13195_2023_1282_MOESM1_ESM.docx]

**Supplementary data**

**Figure S1. Mouse body weight after bilateral ECB implantation in hippocampus.** No significant body weight loss was observed after ECB device bilateral implantation.


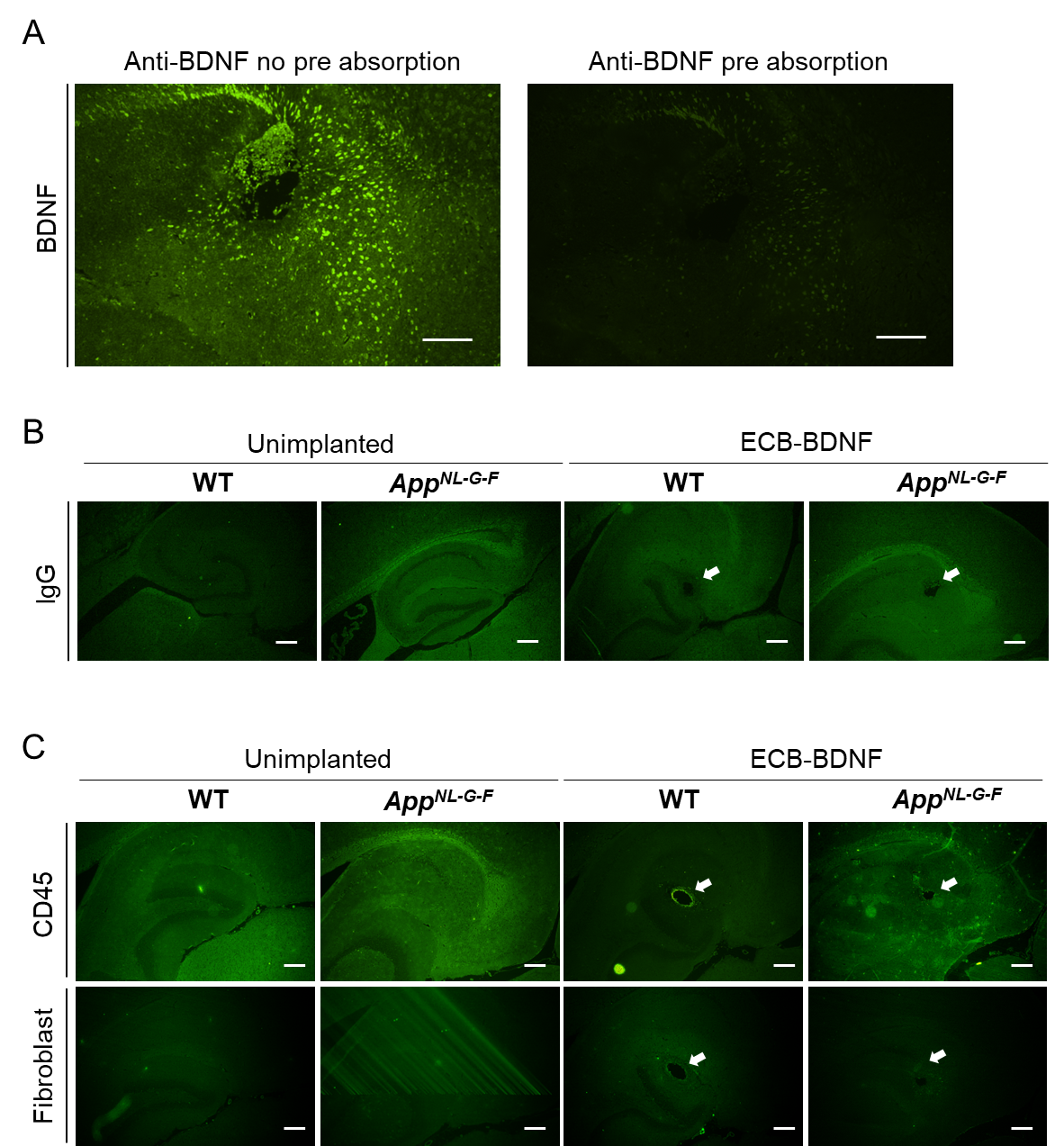


**Figure S2. Specificity of BDNF antibody and immunofluorescence staining for IgG, CD45 and fibroblasts.** (A) Evaluation of BDNF staining without antibody pre-absorption and with antibody absorption with recombinant BDNF protein, shows specificity of the anti-BDNF antibody. (B) Immunofluorescence analysis of IgG with representative staining of brains after one month of ECB-BDNF treatment in cohort 1, indicating no or minimal blood-brain barrier alteration. (C) Representative immunofluorescence staining for CD45 and fibroblast after ECB-BDNF treatment in cohort 2. Scale bar 300 μm.


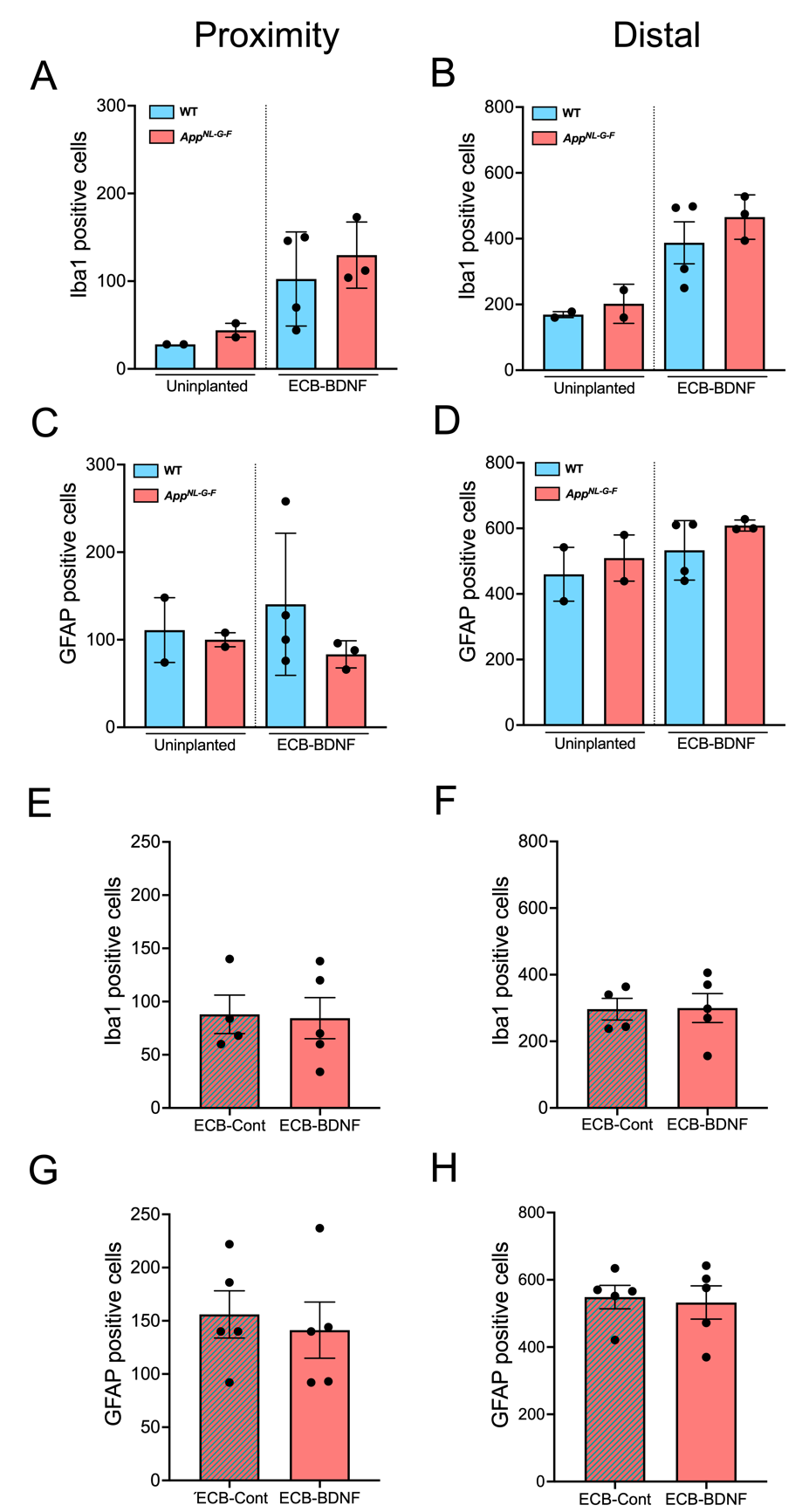


**Figure S3. Immunofluorescence analysis of microglia and astrocyte cells in the proximity and distal area surrounding the implanted ECBs at one-month and four-month post-surgery.** (A,B) Total count of cells positive for the microglia marker Iba1, (C, D) and astrocyte marker GFAP in the proximity and distal area in control (un-implanted) and surrounding the ECB-BDNF devices implanted in WT and *App^NL-G-F^* mice (n = 2 - 4). (E, F) Total cell count positive for the microglia marker Iba1, (G, H) and astrocyte marker GFAP at four-month post-surgery in *App^NL-G-F^* mice in both proximity and distal area surrounding the ECB-Control and ECB-BDNF devices. (n = 2 - 5). In A-D statistical comparison between groups were not applied due to the limited number of animals in control groups. E-H results were analyzed by unpaired Student’s t-test. All data are represented as mean ± S.E.M (See Supplementary table 3).
