# Supplementary material for: Feasibility and therapeutical potential of local intracerebral encapsulated cell biodelivery of BDNF to AppNL−G−F knock-in Alzheimer mice
Source: Alzheimers Res Ther. 2023 Aug 18;15:137. doi: 10.1186/s13195-023-01282-x (PMC10436657; doi:10.1186/s13195-023-01282-x)
Supplement: Supplementary file 2 — Additional file 2: Supplementary Table 1. List of the antibodies. [file 13195_2023_1282_MOESM2_ESM.docx]

**Supplementary table 1.** List of the antibodies

| **Antibody** | **Species Raised** | **Dilution** | **Product Code** | **Source** |
| --- | --- | --- | --- | --- |
| *GFAP* | Rabbit | 1:500 | Z0334 | DAKO |
| *Iba1* | Rabbit | 1:400 | CAK1997 | Wako |
| *BDNF* | Rabbit | 1:500 | AB1534SP | Sigma-Aldrich |
| *82E1* | Mouse | 1:1000 | 10323 | IBL |
| *CD45* | Rabbit | 1:100 | ZRB1180-4X25UL | Sigma-Aldrich |
| *IgG* | Donkey | 1:200 | NL009 | R&D Systems |
| *Fibroblast* | Rat | 1:200 | NB100-64932 | Novus Biologicals |
| *APC/Cy7 anti-mouse B220* | Rat | 1:100 | 103224 | BioLegend |
| *Brilliant Violet 510 anti-mouse CD3e* | Hamster | 1:40 | 100353 | BioLegend |
| *Brilliant Violet 711 anti-mouse TNF-* α | Rat | 1:40 | 506349 | BioLegend |
| *PerCP anti-mouse CD45* | Rat | 1:100 | 557235 | BD Biosciences |
| *Pacific Blue anti-mouse CD11b* | Rat | 1:50 | 101223 | BioLegend |
| *FITC anti-mouse NKp46* | Rat | 1:50 | 137605 | BioLegend |
| *Alexa Fluor 700 anti-mouse CD11c* | Hamster | 1:200 | 117319 | BioLegend |
