# Supplementary material for: Feasibility and therapeutical potential of local intracerebral encapsulated cell biodelivery of BDNF to AppNL−G−F knock-in Alzheimer mice
Source: Alzheimers Res Ther. 2023 Aug 18;15:137. doi: 10.1186/s13195-023-01282-x (PMC10436657; doi:10.1186/s13195-023-01282-x)
Supplement: Supplementary file 3 — Additional file 3: Supplementary Table 2. Data for Fig. 5. [file 13195_2023_1282_MOESM3_ESM.docx]

| **Figue 5A**  **Supplementary Table 2.** Data for figure 5 |  |  |  |  |  | **Figure 5B** |  |  |  |  |
| --- | --- | --- | --- | --- | --- | --- | --- | --- | --- | --- |
|  | WT Control | NLGF Control | WT ECB-BDNF | NLGF ECB-BDNF |  |  | WT Control | NLGF Control | WT ECB-BDNF | NLGF ECB-BDNF |
| Number of values | 2 | 2 | 3 | 3 |  | Number of values | 2 | 2 | 3 | 3 |
|  |  |  |  |  |  |  |  |  |  |  |
| Minimum | 1,906 | 3,265 | 8,604 | 6,933 |  | Minimum | 1,895 | 3,115 | 3,858 | 5,213 |
| Maximum | 2,997 | 4,241 | 20,15 | 31,36 |  | Maximum | 12,01 | 3,71 | 12,87 | 10,45 |
| Range | 1,091 | 0,976 | 11,55 | 24,43 |  | Range | 10,12 | 0,595 | 9,008 | 5,241 |
|  |  |  |  |  |  |  |  |  |  |  |
| **Mean** | **2,452** | **3,753** | **13,46** | **15,68** |  | **Mean** | **6,953** | **3,413** | **8,171** | **8,041** |
| Std. Deviation | 0,7715 | 0,6901 | 5,99 | 13,61 |  | Std. Deviation | 7,152 | 0,4207 | 4,516 | 2,645 |
| Std. Error of Mean | 0,5455 | 0,488 | 3,459 | 7,86 |  | Std. Error of Mean | 5,058 | 0,2975 | 2,607 | 1,527 |
|  |  |  |  |  |  |  |  |  |  |  |
|  |  |  |  |  |  |  |  |  |  |  |
| **Figue 5C** |  |  |  |  |  | **Figure 5D** |  |  |  |  |
|  | WT Control | NLGF Control | WT ECB-BDNF | NLGF ECB-BDNF |  |  | WT Control | NLGF Control | WT ECB-BDNF | NLGF ECB-BDNF |
| Number of values | 2 | 2 | 4 | 3 |  | Number of values | 2 | 2 | 4 | 3 |
|  |  |  |  |  |  |  |  |  |  |  |
| Minimum | 4,782 | 2,572 | 13 | 13,46 |  | Minimum | 4,016 | 2,03 | 6,744 | 5,139 |
| Maximum | 7,256 | 9,756 | 33,08 | 14,7 |  | Maximum | 4,563 | 3,45 | 11,86 | 5,805 |
| Range | 2,474 | 7,184 | 20,08 | 1,236 |  | Range | 0,547 | 1,42 | 5,115 | 0,666 |
|  |  |  |  |  |  |  |  |  |  |  |
| **Mean** | **6,019** | **6,164** | **25,51** | **14,24** |  | **Mean** | **4,29** | **2,74** | **8,701** | **5,446** |
| Std. Deviation | 1,749 | 5,08 | 8,699 | 0,6806 |  | Std. Deviation | 0,3868 | 1,004 | 2,338 | 0,3361 |
| Std. Error of Mean | 1,237 | 3,592 | 4,35 | 0,393 |  | Std. Error of Mean | 0,2735 | 0,71 | 1,169 | 0,1941 |
|  |  |  |  |  |  |  |  |  |  |  |
|  |  |  |  |  |  |  |  |  |  |  |
| **Figue 5E** |  |  |  |  |  | **Figure 5F** |  |  |  |  |
|  | WT Control | NLGF Control | WT ECB-BDNF | NLGF ECB-BDNF |  |  | Wt Control | NLGF Control | WT ECB-BDNF | NLGF ECB-BDNF |
| Number of values | 2 | 2 | 4 | 3 |  | Number of values | 2 | 2 | 4 | 3 |
|  |  |  |  |  |  |  |  |  |  |  |
| Minimum | 8,248 | 13,39 | 18,03 | 18,85 |  | Minimum | 9,849 | 13,99 | 10,94 | 16,48 |
| Maximum | 10,15 | 27,69 | 69,02 | 38,8 |  | Maximum | 14,2 | 24,93 | 25,71 | 19,14 |
| Range | 1,903 | 14,3 | 50,99 | 19,95 |  | Range | 4,347 | 10,93 | 14,76 | 2,659 |
|  |  |  |  |  |  |  |  |  |  |  |
| **Mean** | **9,2** | **20,54** | **46,03** | **28,56** |  | **Mean** | **12,02** | **19,46** | **21,39** | **17,94** |
| Std. Deviation | 1,346 | 10,11 | 21,22 | 9,986 |  | Std. Deviation | 3,074 | 7,73 | 6,989 | 1,348 |
| Std. Error of Mean | 0,9515 | 7,151 | 10,61 | 5,765 |  | Std. Error of Mean | 2,174 | 5,466 | 3,494 | 0,7785 |
|  |  |  |  |  |  |  |  |  |  |  |
|  |  |  |  |  |  |  |  |  |  |  |
| **Figue 5G** |  |  |  |  |  | **Figure 5H** |  |  |  |  |
|  | WT Control | NLGF Control | WT ECB-BDNF | NLGF ECB-BDNF |  |  | WT Control | NLGF Control | WT ECB-BDNF | NLGF ECB-BDNF |
| Number of values | 2 | 2 | 4 | 3 |  | Number of values | 2 | 2 | 4 | 3 |
|  |  |  |  |  |  |  |  |  |  |  |
| Minimum | 0,377 | 1,478 | 6,542 | 20,12 |  | Minimum | 0,572 | 2,703 | 4,644 | 4,834 |
| Maximum | 1,54 | 3,37 | 35,93 | 79,15 |  | Maximum | 1,299 | 3,417 | 7,324 | 16,1 |
| Range | 1,163 | 1,892 | 29,39 | 59,03 |  | Range | 0,727 | 0,714 | 2,68 | 11,27 |
|  |  |  |  |  |  |  |  |  |  |  |
| **Mean** | **0,9585** | **2,424** | **25,46** | **41,04** |  | **Mean** | **0,9355** | **3,06** | **6,544** | **8,817** |
| Std. Deviation | 0,8224 | 1,338 | 13,48 | 33,05 |  | Std. Deviation | 0,5141 | 0,5049 | 1,279 | 6,318 |
| Std. Error of Mean | 0,5815 | 0,946 | 6,739 | 19,08 |  | Std. Error of Mean | 0,3635 | 0,357 | 0,6396 | 3,648 |
|  |  |  |  |  |  |  |  |  |  |  |
|  |  |  |  |  |  |  |  |  |  |  |
| **Figue 5i** |  |  |  |  |  | **Figure 5J** |  |  |  |  |
|  | WT Control | NLGF Control | WT ECB-BDNF | NLGF ECB-BDNF |  |  | WT Control | NLGF Control | WT ECB-BDNF | NLGF ECB-BDNF |
| Number of values | 2 | 2 | 3 | 3 |  | Number of values | 2 | 2 | 3 | 3 |
|  |  |  |  |  |  |  |  |  |  |  |
| Minimum | 0,116 | 0,026 | 7,052 | 3,777 |  | Minimum | 0,328 | 0,328 | 2,972 | 1,9 |
| Maximum | 1,475 | 2,37 | 43,45 | 10,88 |  | Maximum | 1,211 | 1,644 | 7,522 | 2,799 |
| Range | 1,359 | 2,344 | 36,4 | 7,101 |  | Range | 0,883 | 1,316 | 4,55 | 0,899 |
|  |  |  |  |  |  |  |  |  |  |  |
| **Mean** | **0,7955** | **1,198** | **19,86** | **7,129** |  | **Mean** | **0,7695** | **0,986** | **4,85** | **2,426** |
| Std. Deviation | 0,961 | 1,657 | 20,46 | 3,567 |  | Std. Deviation | 0,6244 | 0,9306 | 2,377 | 0,4688 |
| Std. Error of Mean | 0,6795 | 1,172 | 11,81 | 2,06 |  | Std. Error of Mean | 0,4415 | 0,658 | 1,372 | 0,2707 |
