# Supplementary material for: Feasibility and therapeutical potential of local intracerebral encapsulated cell biodelivery of BDNF to AppNL−G−F knock-in Alzheimer mice
Source: Alzheimers Res Ther. 2023 Aug 18;15:137. doi: 10.1186/s13195-023-01282-x (PMC10436657; doi:10.1186/s13195-023-01282-x)
Supplement: Supplementary file 4 — Additional file 4: Supplementary Table 3. Data for Supplementary Fig. 3. [file 13195_2023_1282_MOESM4_ESM.docx]

| **Figure S3A**  **Supplementary Table 3**. Data for Supplementary Figure 3 |  |  |  |  |  | **Figure S3B** |  |  |  | |  | |
| --- | --- | --- | --- | --- | --- | --- | --- | --- | --- | --- | --- | --- |
|  | WT Control | NLGF Control | WT ECB-BDNF | NLGF  ECB-BDNF |  |  | Wt Control | NLGF Control | WT  ECB-BDNF | | NLGF  ECB-BDNF | |
| Number of values | 2 | 2 | 4 | 3 |  | Number of values | 2 | 2 | 4 | | 3 | |
|  |  |  |  |  |  |  |  |  |  | |  | |
| Minimum | 28 | 36 | 44 | 104 |  | Minimum | 160 | 160 | 250 | | 394 | |
| Maximum | 28 | 52 | 150 | 173 |  | Maximum | 178 | 244 | 498 | | 528 | |
| Range | 0 | 16 | 106 | 69 |  | Range | 18 | 84 | 248 | | 134 | |
|  |  |  |  |  |  |  |  |  |  | |  | |
| Mean | 28 | 44 | 102,5 | 129,7 |  | Mean | 169 | 202 | 387,5 | | 465,7 | |
| Std. Deviation | 0 | 11,31 | 53,63 | 37,74 |  | Std. Deviation | 12,73 | 59,4 | 127,5 | | 67,49 | |
| Std. Error of Mean | 0 | 8 | 26,81 | 21,79 |  | Std. Error of Mean | 9 | 42 | 63,76 | | 38,96 | |
|  |  |  |  |  |  |  |  |  |  | |  | |
|  |  |  |  |  |  |  |  |  |  | |  | |
| **Figure S3C** |  |  |  |  |  | **Figure S3D** |  |  |  | |  | |
|  | WT Control | NLGF Control | WT ECB-BDNF | NLGF ECB-BDNF |  |  | WT Control | NLGF Control | WT ECB-BDNF | NLGF ECB-BDNF | |  |
| Number of values | 2 | 2 | 4 | 3 |  | Number of values | 2 | 2 | 4 | | 3 | |
|  |  |  |  |  |  |  |  |  |  | |  | |
| Minimum | 74 | 92 | 76 | 66 |  | Minimum | 378 | 439 | 440 | | 598 | |
| Maximum | 148 | 108 | 258 | 96 |  | Maximum | 542 | 580 | 612 | | 628 | |
| Range | 74 | 16 | 182 | 30 |  | Range | 164 | 141 | 172 | | 30 | |
|  |  |  |  |  |  |  |  |  |  | |  | |
| Mean | 111 | 100 | 140,5 | 83,33 |  | Mean | 460 | 509,5 | 533 | | 608,7 | |
| Std. Deviation | 52,33 | 11,31 | 81,16 | 15,53 |  | Std. Deviation | 116 | 99,7 | 90,9 | | 16,77 | |
| Std. Error of Mean | 37 | 8 | 40,58 | 8,969 |  | Std. Error of Mean | 82 | 70,5 | 45,45 | | 9,684 | |
|  |  |  |  |  |  |  |  |  |  | |  | |
|  |  |  |  |  |  |  |  |  |  | |  | |
| **Figure S3E** |  |  |  |  |  | **Figure S3F** |  |  |  | |  | |
|  |  |  | Iba1 4 MONTHS BDNF Proximity | |  |  |  |  | Iba1 4 MONTHS BDNF Distal | | | |
| Column B |  |  | ECB-BDNF |  |  | Column B |  |  | ECB-BDNF | |  | |
| vs. |  |  | vs, |  |  | vs. |  |  | vs, | |  | |
| Column A |  |  | ECB-Cont |  |  | Column A |  |  | ECB-Cont | |  | |
|  |  |  |  |  |  |  |  |  |  | |  | |
| Unpaired t test |  |  |  |  |  | Unpaired t test |  |  |  | |  | |
| P value |  |  | 0,8979 |  |  | P value |  |  | 0,9527 | |  | |
| P value summary |  |  | ns |  |  | P value summary |  |  | ns | |  | |
| Significantly different (P < 0.05)? | | | No |  |  | Significantly different (P < 0.05)? | | | No | |  | |
| One- or two-tailed P value? | |  | Two-tailed |  |  | One- or two-tailed P value? | |  | Two-tailed | |  | |
| t, df |  |  | t=0,1331, df=7 |  |  | t, df |  |  | t=0,06149, df=7 | |  | |
|  |  |  |  |  |  |  |  |  |  | |  | |
|  |  |  |  |  |  |  |  |  |  | |  | |
| **Figure S3G** |  |  |  |  |  | **Figure S3H** |  |  |  | |  | |
|  |  |  | GFAP 4 MONTHS BDNF Proximity | |  |  |  |  | GFAP 4 MONTHS BDNF Distal | | | |
| Column B |  |  | ECB-BDNF |  |  | Column B |  |  | ECB-BDNF | |  | |
| vs. |  |  | vs, |  |  | vs. |  |  | vs, | |  | |
| Column A |  |  | *´*ECB-Cont |  |  | Column A |  |  | ECB-Cont | |  | |
|  |  |  |  |  |  |  |  |  |  | |  | |
| Unpaired t test |  |  |  |  |  | Unpaired t test |  |  |  | |  | |
| P value |  |  | 0,6792 |  |  | P value |  |  | 0,7982 | |  | |
| P value summary |  |  | ns |  |  | P value summary |  |  | ns | |  | |
| Significantly different (P < 0.05)? | | | No |  |  | Significantly different (P < 0.05)? | | | No | |  | |
| One- or two-tailed P value? | |  | Two-tailed |  |  | One- or two-tailed P value? | |  | Two-tailed | |  | |
| t, df |  |  | t=0,4291, df=8 |  |  | t, df |  |  | t=0,2644, df=8 | |  | |
